# What is needed to sustain improvements in hospital practices post-COVID-19? a qualitative study of interprofessional dissonance in hospital infection prevention and control

**DOI:** 10.1186/s12913-022-07801-0

**Published:** 2022-04-14

**Authors:** Gwendolyn L. Gilbert, Ian Kerridge

**Affiliations:** 1 Marie Bashir Institute for Infectious Diseases and Biosecurity, Westmead Institute for Medical Research, 176 Hawkesbury Rd, Westmead, NSW 2145 Australia; 2 Sydney Health Ethics, School of Public Health, The University of Sydney, Edward Ford Building (A27) Fisher Road, Sydney, NSW 2006 Australia; 3 Department of Haematology, Royal North Shore Hospital, Reserve Rd, St Leonards, St Leonards, NSW 2065 Australia

**Keywords:** Infection prevention and control, Doctor-nurse game, Healthcare-worker infections, COVID-19, Bio preparedness, Professional ethics

## Abstract

**Background:**

Hospital infection prevention and control (IPC) depends on consistent practice to achieve its purpose. Standard precautions are embedded in modern healthcare policies, but not uniformly observed by all clinicians. Well-documented differences in attitudes to IPC, between doctors and nurses, contribute to suboptimal IPC practices and persistence of preventable healthcare-associated infections. The COVID-19 pandemic has seriously affected healthcare professionals’ work-practices, lives and health and increased awareness and observance of IPC. Successful transition of health services to a ‘post-COVID-19’ future, will depend on sustainable integration of lessons learnt into routine practice.

**Methods:**

The aim of this pre-COVID-19 qualitative study was to investigate factors influencing doctors’ IPC attitudes and practices, whether they differ from those of nurses and, if so, how this affects interprofessional relationships. We hypothesised that better understanding would guide new strategies to achieve more effective IPC. We interviewed 26 senior clinicians (16 doctors and 10 nurses) from a range of specialties, at a large Australian tertiary hospital. Interview transcripts were reviewed iteratively, and themes identified inductively, using reflexive thematic analysis.

**Results:**

Participants from both professions painted clichéd portraits of ‘typical’ doctors and nurses and recounted unflattering anecdotes of their IPC behaviours. Doctors were described as self-directed and often unaware or disdainful of IPC rules; while nurses were portrayed as slavishly following rules, ostensibly to protect patients, irrespective of risk or evidence. Many participants believed that doctors object to being reminded of IPC requirements by nurses, despite many senior doctors having limited knowledge of correct IPC practice. Overall, participants’ comments suggested that the ‘doctor-nurse game’—described in the 1960s, to exemplify the complex power disparity between professions—is still in play, despite changes in both professions, in the interim.

**Conclusions:**

The results suggest that interprofessional differences and inconsistencies constrain IPC practice improvement. IPC inconsistencies and failures can be catastrophic, but the common threat of COVID-19 has promoted focus and unity. Appropriate implementation of IPC policies should be context-specific and respect the needs and expertise of all stakeholders. We propose an ethical framework to guide interprofessional collaboration in establishing a path towards sustained improvements in IPC and bio-preparedness.

## Introduction

The basic principles of hospital infection prevention and control (IPC) – including hand hygiene, sanitation and environmental hygiene, fresh air and appropriate use of personal protective equipment—were established and gradually adopted into practice in the nineteenth century. In the twenty-first century, standard and transmission-based precautions are accepted as routine health care practices, designed to minimise rates of preventable healthcare associated infection (HAI) and transmission of antimicrobial resistance (AMR) [1–3]. Despite this, HAIs continue to challenge health services and compromise patient outcomes [4]. A recent modelling study estimated that, in 2016/17, HAIs affected ~ 5% of adult inpatients and ~ 2% of frontline healthcare workers (HCWs) in National Health Service (NHS) hospitals in England. They resulted in the deaths of ~ 28,500 patients. The estimated cost to the NHS, of the associated loss of 7.1 million occupied bed days (21% of the total) and 79,700 days’ absenteeism among frontline HCWs, was £2.7 billion [5]. A systematic review and metanalysis, of interventions designed to reduce the rates of selected HAIs, indicated that 35–55% of serious HAIs would be preventable if accepted IPC measures were applied consistently [6]. However, other studies have found that optimal IPC practices often fail because of, *inter alia*, organisational, systemic and behavioural factors [7, 8].

HAI and AMR transmission rates are poorly monitored in Australia [9] and often underestimated, but exotic or emerging infectious diseases (EIDs) that threaten the health or lives of HCWs are hard to ignore. A series of twenty-first century EIDs, including SARS, MERS, and Ebola virus disease (EVD), [10–12] focused attention on hospital IPC and changed HCW behaviour, albeit in relatively few countries and/or temporarily [13, 14]. The effects on hospital patients and HCWs, of COVID-19, have been global [15] and will be prolonged; but they vary, even allowing for differences in community transmission and incomplete data [16]. In many countries, inadequate health system preparedness and shortages of personal protective equipment (PPE) have been blamed for the high burden of HCW deaths [14, 17, 18]. In other countries’ nosocomial HCW infections have been largely prevented by pre-emptive escalation and consistent application of IPC measures, despite high levels of exposure to COVID-19 [19–21].

Now that many countries in the developed world are considering a post-COVID-19 future, protected by widespread immunisation, there is some urgency to ensure that the lessons are consolidated. A comparison of pre-COVID-19 IPC practices—and the professional values and organisational policies that influenced them—with those introduced during the pandemic, will help to inform decisions about which can be relaxed when the immediate danger wanes, and which should become business-as-usual, to ensure that healthcare sector is not caught unawares by the next pandemic.

## Background

IPC practices are influenced by, inter alia*,* historical, professional, and cultural factors that had their origins in the nineteenth century. By mid-century medical science had progressed to a point where doctors could, significantly, improve patient outcomes; but more complex medical and surgical remedies needed skilled nurses, to support them [22]. Initially nurses were trained and supervised by doctors but, as more educated middle-class women became nurses, they demanded more professional independence and recognition. Relations between the powerful medical establishment and the emerging nursing profession, were characterised by conflict over roles and status, complicated by gender, class, religion, politics, and educational differences [23].

The promise of medical and surgical advances was limited by appalling hospital mortality rates due to infection, until improvements in hospital hygiene and design – often driven by nurses—were gradually implemented, and rates began to fall towards the end of the nineteenth century. Vaccines and antibiotics contributed to further improvement in the early twentieth century, but increasing antibiotic resistance and a dwindling supply of new agents, provided the impetus for revival of hospital IPC programmes in the 1960s-70 s [24, 25]. IPC is now established in hospital practice, supported by a growing body of robust evidence, [26, 27] but there are wide variations in resources and effectiveness [28–30]. Antibiotics are no longer a reliable panacea, [31] and hospitals are still subject to regular outbreaks of viral infection, [32] which now include COVID-19 [15, 33].

As a group, doctors are, generally, less observant of accepted IPC practices than other health professionals [8, 34–37]. In Australia, this is reflected in rates of compliance with the ‘5 moments of hand hygiene’, the only IPC practice that is systematically monitored in public hospitals. Doctors’ compliance has been consistently 10–15% lower than that of nurses since national reporting began in 2009 [38].

In this paper, we describe the results of a qualitative study, conducted before the start of the COVID-19 pandemic in a large tertiary hospital, in which we sought the views of senior clinicians about the professional and cultural factors that influence doctors’ attitudes to, and practice of IPC, whether they differed from those of nurses and, if so, how this affected interprofessional relationships. We focused on doctors, primarily, because we believe that their status in the hospital means that they disproportionately influence organisational culture, priorities and, indirectly, IPC practices. Based on the results, we propose strategies that may improve interprofessional co-operation and, thereby, sustain COVID-19 driven improvements in IPC.

## Methods

This was a qualitative case study of a large (~ 950-bed) university teaching hospital in Sydney, Australia, which provides a full range of specialist services to more than 90,000 inpatients, annually. The aim was to provide a detailed understanding of aspects of IPC at a single site, without the potential confounding effects of different policies, patient populations and physical environments.

### Researchers’ backgrounds and relationship to participants

Both researchers are senior physicians with a special interest in hospital IPC, based on longstanding experience of caring for patients, who have suffered from preventable HAIs and/or the uncertainty and fear associated with becoming colonised by a multidrug-resistant organism. We were employed, before and/or during this study, at the hospital where it was conducted, as staff specialists in infectious diseases/clinical microbiology (GLG) or haematology/bone marrow transplantation (IK) and known to most participants but, with few exceptions, had not worked closely with them.

### Participant selection

Participants were senior medical (*N* = 16) and nursing (*N* = 10) clinicians and/or managers or directors, with 10–40 years’ specialist experience (summarised in Table 1). They were purposively selected, based on their senior positions and/or the researchers’ knowledge of them, as likely to have a broad experience and perspective of hospital doctors’ attitudes and practices and to be willing to express their views openly to a senior colleague. Our decision to include only senior clinicians was confirmed by the Human Research Ethics Committee (HREC) reviewers who were concerned that junior clinicians may be intimidated by, and unwilling to speak frankly to, a senior consultant as interviewer. Medical participants were full-time senior staff specialists [SS] or visiting medical officers (VMOs), contracted on a sessional basis; nurses were all full-time hospital employees in leadership positions.

**Table 1:** Characteristics of participants

	**Docto**r**s**^**a**^** (*N* = 16)**		**Nurses (*N* = 10)**	
Gender M: F	10:6		3:7	
Role	Senior executive/ divisional director (MDD)	6	Divisional director/deputy; or facility manager (NDD)	6
	Medical unit/departmental director (MUD)	4	Nursing unit manager (NUM)	2
	Senior medical consultant (SMC)	6	Clinical nurse consultant (CNC)	2
Specialty	Medical 6; surgical 4; other 6^b^	16	Medical 5; surgical 3; other 2^c^	10

^a^Staff specialists (SS), 11; visiting medical officers (VMO). 5

^b^Other doctors’ specialties: intensive care, 2; emergency medicine, 2; anesthetics,1; obstetrics/gynecology, 1

^c^Other nurses’ specialties: intensive care, 1; infection prevention and control, 1

### Data collection

Semi-structured interviews were guided by a small number of open-ended questions (Table 2). Participants were not specifically asked for their views of nurses’ IPC practices or how they differed from those of doctors, initially, but many offered opinions, spontaneously. Interviews were recorded and transcribed by a professional transcription service, with participants’ informed consent.

**Table 2:** Interview topics/questions used to prompt discussion with participants

1. To what extent do doctors, generally, regard healthcare associated infections (HAIs) as a significant problem in this hospital? Could some/most HAIs be prevented? How?
2. The literature suggests that doctors generally comply less consistently than nurses with infection prevention and control (IPC) measures. Is this consistent with your experience? Why is it so? Does it adversely affect patient care?
3. What changes, if any, would you make in hospital IPC policies to make them more acceptable to doctors, increase adherence and reduce risks to patients?

### Data analysis

Transcripts were reviewed and coded independently by both authors, using a reflexive thematic analysis approach [39]. We chose this approach on the assumption that our experience at the study hospital, knowledge of its culture and history and personal acquaintance with participants would inevitably influence our interpretation of data and therefore require us to continuously review and question our findings.

We identified broad-brush themes, inductively, based on patterns of meaning. As codes were added, subthemes were developed and analysed, iteratively, in relation to research aims and interpreted in the context of our own experiences as senior clinicians. We also noted participants’ rhetorical styles that reflected emotional responses to these issues.

In reporting our findings, we designated participants’ professions and roles broadly, to preserve anonymity, as: nursing divisional director (NDD), nursing unit manager (NUM), clinical nurse consultant (CNC); medical divisional director (MDD), medical unit director (MUD), senior medical consultant (SMC).

## Findings

 Our initial analysis identified four broad-brush themes (Table 3). Theme 1 has been described previously [40]. Within Theme 2, we identified four subthemes, which are subject of this paper. Table 4 shows representative quotes, illustrating subthemes.

**Table 3:** Factors affecting attitudes to and practices of infection prevention and control (IPC) – themes identified by transcript analysis

1. Characteristics of doctors, medical culture, and medical professionalism^a^
2. Interprofessional influences on attitudes to and practices of IPC
a) Professional characteristics—stereotypes
b) Unflattering, but varied, interprofessional perceptions
c) Doctors’ reluctance to accept advice from expert nurse consultants
d) The “doctor-nurse game”
3. Organisational factors affecting IPC practices
4. Factors relating to IPC policies and/or their implementation

^a^Previously reported [40]

**Table 4:** Illustrative participants’ quotes (Q) describing inter-/intra-professional differences in attitudes to and practice of infection prevention and control (IPC)

**Professional characteristics—stereotypes**	
Q4:1 A medical divisional director’s (MDD1) description of nursing and medical stereotypes	Nurses are very process driven. They have very … hierarchical structures that they stick rigidly within. If they drift outside they get jumped upon, they eat their young… Most of the sort of people that go into medicine are more independent thinkers and they don't like to be told what to do by anyone and the further up the tree you go the more like that you become… [they have] a view about themselves that … they're above criticism
Q4:2. A nursing divisional director’s (NDD1) different view of stereotypes	I think the nurses are very keen to do the right thing… to the best of their ability.… it's disheartening when you see others coming in and out of rooms and not doing that and… when you [speak] to them there's a stand-up argument about hand hygiene
Q4:3. An IPC nurse clinical consultant (CNC1) blames rigid application of rules for doctors’ lack of respect for IPC	I think medical staff are really driven by evidence, the fact that contextually they can see… validity. So if they think something's stupid… and they’re just doing it because it's [the rule] … they lose respect for it. … there's a lot of infection control procedures like that. … Whereas the nurses are more accepting of tradition—that's the way we've done it because… Florence has done it… And they're not looking as much for that evidence
**Unflattering, but varied interprofessional perceptions**	
Q4:4. MDD2 suggests bias on the part of ward auditors	The audits say that the doctors are terrible, but most of the audits are done by the nurses. So you wonder whether there’s a bit of payback. …it’s Schadenfreude, isn’t it? Maybe they feel they’re kicked around by doctors all the time. It’s nice to point out that the doctors aren’t perfect
Q4:5. MDD4 suggests bossy nurses as hand hygiene leaders	Who is leading? … the nurses would be much better [than doctors] at bossing people around about hand washing, because they’re pretty good at bossing you around about everything else
Q4:6. NDD1 speaks of NUMs’ problem with doctors’ poor hand hygiene compliance	I'd look at my rates across the wards [and ask the NUM] ‘Your rates are sitting below the benchmark, what are you doing about that in your ward?’ I would get back: ‘When I break it down… the medical staff sit quite low and …they’re not my responsibility so I can’t impact them’
Q4:7. NDD2 on nurses varied approaches to and responses from medical teams about IPC practices	When I was a NUM, I had a very good relationship with the team so I could say to them ‘Hey, you haven’t even washed your hands’ and they would listen. [Other] people don't feel that they can say that, or they’ve got 10 teams coming through and it's very hard to build relationships…. So if you pull someone up they’re going, ‘Oh, don't worry about it, we’ll just move on, let’s get through this and don't worry about her’
Q4:8. MDD1 admits doctors are ignorant	Some feedback clinicians get sounds a bit like they’re deliberately doing people harm—most of it is acts of ignorance
Q4:9. A nursing unit manager (NUM1) describes poor IPC attitudes & practice in a surgical unit	The infection control team were just flabbergasted. They said ‘They didn't wash their hands at all, not once that whole ward round. They were touching open wounds and then going off and touching the next patient.’ …[the MUD].. was livid, but, not at his team. He was livid at us that we did this audit [and that it was] was out there for everyone to see
**Doctors reluctance to accept advice from expert nurse consultants**	
Q4:10. CNC1 on surgeons’ response to high surgical site infection rates	The only feedback we’ve had was that … [our hospital] was one of the worst-performing globally. …they [surgeons] engaged [infectious diseases physician] to look at their strategies…. [who] came to us for information, [but] there’s a lot …being missed just based on her length of experience
Q4:11. MDD2 on senior doctors’ response to Ebola preparations	I thought the intensivists’ behavior, rather than dampening fears, exaggerated them… nursing staff behaved far better..[although] they were far greater at risk. …there was great debate about the reliability and validity of the [IPC unit’s] advice….Professor Google became problematic.”
**The “doctor-nurse game”**	
Q4:12. A medical consultant on nurses’ approaches to doctors	Junior doctors, don’t mind being told what to do, but only in a way that’s appropriate… So [a nurse] who comes along and says you have to do these things…there’s no flexibility … that’s always going to meet resistance.”
Q4:13. MDD4 recounts junior doctors’ complaints that nurses interpret policy too literally	Residents …were saying that some wards will take [peripheral intravenous cannulas] out – ‘oh, it’s 72 h, we’ll whip it out’—they haven’t looked at it; they haven’t looked to see when the next [antibiotic] dose is, and they call you, and you have 15 other calls that shift… they’re not equally shouldering the responsibility.”

### Professional stereotypes

Participants’ comments reflected conventional stereotypes of nurses’ and doctors’ professional ‘personalities’. Doctors were characterised (by participants of both professions) as ‘independent thinkers’ (Table 4, Quote 4:1), focused on diagnosis and treatment of individual patients, relying on personal judgment, indifferent to organisational policies and driven by ambition. By contrast, nurses were characterised (mainly by doctors) as driven by rules, rigidly enforced by the nursing hierarchy (Q4:1).

Nurses were said to be more aware of infection risks than doctors because they spend more time with patients and feel obliged to ‘work to policy’ to ensure a safe environment (Q4:2). However, some participants gave examples of inflexible application of rules that they believed could undermine doctors’ respect for policies (Q4:3) and lead to unsafe decisions. Examples included: a) a patient with mild post-chemotherapy fever, being placed in isolation, in the only available single room – according to ward policy—while a patient with acute respiratory infection and cough, remained in the open ward, exposing immunocompromised patients to a risk of infection (MUD1); b) a teenager with lymphoma, identified by routine screening as a carrier of vancomycin resistant enterococci being placed in prolonged isolation, with significant adverse psychological effects, despite posing minimal risk to other patients (CNC1).

Several participants commented that doctors often seemed oblivious to IPC rules during ward rounds. A nurse suggested that this was because they were focused on patients’ test results and treatment plans. However, several doctors believed many of their senior colleagues did not know what the IPC policies were, because they avoided mandatory training with impunity. A MDD believed that VMOs would not undertake training because they were not paid to do so, unlike nurses who regularly receive in-service IPC training, from “supernumerary” clinical nurse educators. This MDD thought that doctors would not accept instruction from nurses and suggested they be paid to attend IPC training, presented by a medical specialist. Several participants believed doctors would only adhere to policies if offered convincing evidence of their effectiveness (Q4:3).

### Doctors’ and nurses’ unflattering perceptions of each other

Participants’ attitudes towards members of the ‘other’ profession, revealed significant prejudice. MDD2 suggested that hand hygiene audits were biased against doctors because the auditors were nurses (Q4:4); MDD4 felt that “bossy” nurses should be expected to remind doctors when hand hygiene was required to ensure they complied (Q4:5). NDD1 observed that doctors’ poor compliance reflected unfairly on NUMs, who were accountable for their ward’s audit results (Q4:6).

As previously reported, participants recounted instances of doctors’ rudeness or bullying, in response to reminders about IPC precautions [40]. CNC2 reported asking a consultant to put on a gown before entering the room of an ICU patient who was in isolation, to which he retorted “I don’t care about your bloody infection control” and went in without any precautions. Although outright rudeness was not common, doctors were described as often dismissive or grudgingly compliant when reminded about IPC (Q4:7). MDD1 felt criticism of doctors for noncompliance was unfair since many did not know what was required (Q4:8).

Doctors’ attitudes to IPC may vary even within the same environment. NUM1 described two surgical units that shared the same ward: whereas one unit’s IPC practices were “exemplary”, the other’s consultants rarely visited the ward and its junior doctors consistently ignored nurses’ reminders about standard precautions. After informing these junior doctors of his intention, NUM1 asked the IPC team to audit the team’s hand hygiene compliance during a ward round. He showed the results (zero compliance), to the unit director, whose reaction NUM1 described as “livid” (Q4:9), not at the results, but that the audit had been performed.

Several nurses cited units whose IPC practices they regarded as excellent; their common feature was that the consultants visited the ward regularly, they observed IPC precautions routinely themselves, and made it clear that they expected their teams to do likewise. NDD1 described one surgical unit’s regular ‘structured, interdisciplinary bedside rounds’ in which nurses, allied health professionals, junior doctors, patients and relatives, if present, were included in bedside discussions. Several participants of both professions, implied that senior consultants’ respect for patients and other health professionals correlated with excellent IPC practices. In contrast, some units were identified by several participants as being notorious for their poor IPC practices and (reputedly) high infection rates (based on anecdotes, rather than surveillance data).

### Doctors’ disregard for IPC professionals’ experience and expertise

CNC1 recounted the IPC unit’s prolonged efforts to achieve recognition of their expertise and effectiveness. It was only after the hospital’s poor surveillance results attracted regulatory attention, that the hospital executive began to regularly seek their advice and include them in high level committees. However, they were still often bypassed. CNC1 cited the example of a surgical director seeking advice from a recently appointed medical specialist, rather than an experienced IPC professional, about how to respond to poor results in an international surveillance program (Q4:10). By contrast, IPC unit CNCs were regularly invited to join state and national committees planning responses to infectious disease threats (e.g. SARS, 2003; pandemic influenza H1N1, 2009; EVD, 2014–16), and led the state’s preparations to receive patients with a high consequence infectious disease. MDD2 recalled that, during the EVD threat, some senior doctors sought to overrule the IPC unit’s guidance on PPE use, by demanding excessive, unnecessarily strict precautions. Their demands were rejected, but caused confusion and fear among staff and undermined the IPC team’s authority (Q4:11).

### The doctor-nurse game

We interpreted some participants’ anecdotes as modern examples of the doctor-nurse game, described by Leonard Stein, in 1967 [41], to explain how nurses and doctors were expected to interact, professionally. Stein described a hierarchical relationship, in which doctors were unequivocally superior. Nurses were expected to be resourceful and show initiative but, under no circumstances, to appear to disagree or argue with doctors. Their recommendations about patient care must be made in such a way as to appear to have been initiated by the doctor, Participants described doctors’ complying with IPC policies if a nurse reminded them respectfully; but if the reminder implied criticism or was abrupt or inflexible, it was likely to be ignored or met with an angry or abusive response. See Q4:7, Q4:12 and Q4:13 and (Tables 5 and 6).

**Table 5:** Doctor-nurse game: interprofessional non-communication

MUD1 described his decision to have unit registrars perform hand hygiene audits during medical rounds, and the subsequent improvement in doctors’ hand hygiene compliance. However, he noticed that, when the results were presented at weekly interdisciplinary meetings, senior nurses “seemed to be getting a bit grumpy”; when asked why, the NUM explained that ward auditors had been auditing hand hygiene for months, using standardised methods prescribed by Hand Hygiene Australia (https://www.hha.org.au/). MUD1 was irritated that nurses were “reporting them somewhere but not to us” and that “it took [them] a while to realise this [duplication] doesn’t make a lot of sense”. He regarded the ward auditors’ methods as unnecessarily complex strict and was sceptical of results showing consistently lower compliance among doctors than nurses.

**Table 6:** Doctor-nurse game: interprofessional boundary disputes

Participants mentioned ongoing problems with peripheral intravenous cannula (PIVC) insertions by junior doctors that had contributed to a high Staphylococcus aureus bacteraemia rate. A recently introduced PIVC policy included that: wards provide trolleys stocked with all necessary equipment; date and time of insertion be documented in the patient’s record; and cannulas be replaced after 72 h if still required. MDD4 reported interns’ complaints that: a) nurses often called them, to replace cannulas after hours, having removed them exactly 72 h after insertion (Table 3:13); b) many wards did not have properly stocked trolleys (although NUM1 claimed interns refused to use his ward’s trolleys); nurses claimed to be too busy to complete the documentation.	

### Participants’ emotional rhetorical styles

Participants’ language suggested emotional responses to IPC: e.g. a doctor having “a stand up argument about hand hygiene” (Q4:2); a consultant’s contempt for “your bloody infection control”; a unit director being “livid” about a hand hygiene audit, but indifferent to neglect of hand hygiene (Q4:9); nurses trying “to do the right thing” but “disheartened” by doctors’ behaviour (Q4:2). On the other hand, doctors described how senior nurses “eat their young” (Q4:1) if they broke the rules; and doctors’ poor hand hygiene compliance as nurses’ “payback” for being “kicked around by doctors” (Q4:4).

## Discussion

Participants’ accounts of doctors’ and nurses’ attitudes to IPC and perceptions of each other, support the view of Irvine et al., that “Interprofessional relationships … are frequently distorted by mutual suspicion, hostility and disparities between the way that a particular profession views itself and how it is viewed by other occupations.” [42]. They reflect the persistent influence of professional stereotypes, on interprofessional interactions. Medical and nursing stereotypes, as depicted by participants of both professions, were highly stylised and unflattering. Doctors are focused on diagnosis and “cure”; they prioritise clinical autonomy and object to rules or advice from other professionals that might challenge their decisions; they are ambitious and competitive in pursuit of career goals; they support each other and ignore or seek to dominate other healthcare professionals. Nurses are driven by rules, which they implement rigidly, without concern for evidence or possible inconvenience to doctors; they are motivated by fear of the nursing hierarchy, as well as being “bossy” and vindictive. Nurses know better than doctors, what is best for patients, because they spend more time with them.

Participants acknowledged wide variation in interprofessional interactions, depending on context, unit/specialty culture and personalities. Nevertheless, repeated references to professional stereotypes suggest that the “doctor-nurse game” is still current, albeit in modified form [41, 43]. According to Stein “…nurses were to make recommendations, but their recommendations had to appear to be initiated by the doctor.” In 1990, he suggested the “game” had all but ceased because of changes in professional education, roles and gender ratios [43]. However, persistence of this “dance of deference” has been recently implicated in communication failures, blame-shifting and mutual mistrust, causing harm to patients [44–46]. Our findings suggest that the ‘rules of the game’ mean that a nurse, who seeks to remind a doctor about an IPC breach, must balance the likelihood of success (the doctor’s compliance) vs failure (being ignored or humiliated) and act accordingly.

Compared with other threats to patient safety, such as medication error or wrong-site surgery, an IPC breach may seem a low risk, albeit with potentially high consequences. Because of the low risk/high stakes nature of IPC breaches it is sometimes argued that evidence-based “rules”, should be enforced, using the logic applied to aviation safety [47]. Against this, it is argued that medical practice is too varied and unpredictable to be driven by rules, and idiosyncratic IPC practices are excused on the basis that each patient encounter is unique. However, doctors—unlike airline pilots—often discount risks to ‘customers’ and rarely perceive a personal risk of harm or censure if rules are breached [40]. Moreover, many are unwilling to “abandon historical and cultural precedents and beliefs that are linked to performance and autonomy” [48]. However, preventing serious HAIs requires consistent application of standard precautions. This does not imply either mindless compliance with inflexible rules (nurse stereotype) or a ‘doctor knows best’, laissez-faire approach (medical stereotype) to IPC. Rules should be appropriately and safely modified, according to context and with agreement of relevant stakeholders, but doctors – or anyone—flouting them, without explanation, causes confusion and conflict, which inevitably detracts from patient care.

In 2020 – and not for the first time—the low risk/high stakes IPC balance changed. The fact that failure to isolate an infectious patient or use appropriate PPE can initiate a devastating hospital outbreak, affecting HCWs as well as patients, was demonstrated during previous coronavirus outbreaks [49, 50] and has gained even greater salience during the current pandemic [51]. A lack of confidence and consistency in routine and outbreak-level IPC practice [52, 53] among some HCWs exacerbates the fear associated with a novel threat, even if – like EVD in 2015 – it fails to eventuate [54, 55]. During the current pandemic, high rates of HCW infection and deaths, globally, and perceived deficiencies in health authorities’ preparedness and responses, have engendered distrust [56, 57]. HCWs, especially doctors, have demanded greater safeguards than recommended by international and local experts, based on the precautionary principle [58]. Like other aspects of patient safety, IPC is “not simply a technical issue, but a site of organizational and professional politics” [59].

There are still many aspects of COVID-19 and its control that remain uncertain, but it has undoubtedly raised awareness of IPC among frontline HCWs, hospital administrators and the public. This provides an opportunity for long-term improvements based on lessons learnt, if the tendency to revert to old habits, as the risks fade, can be resisted. Aiming for universal consensus in all aspects of IPC, especially during an emergency, may be unrealistic, but the pandemic has highlighted the dangers of confusion, inconsistency, and lack of trust. Nevertheless, different beliefs about risk, what counts as safe practice and who should decide, militated against HCWs speaking up to a colleague about a potential IPC breach, in the past, especially if they feared a hostile or dismissive response [60–62]. The fact that their fear was justified was reflected in language used by participants to describe what they saw as some doctors’ petty or aggressive behaviour [40]. However, the use of ‘spotters’ to monitor use of PPE and other IPC practices, during the COVID-19 pandemic, has been well-received by HCWs and effective in increasing compliance and reducing HCW infections [63].

Under most circumstances, IPC policies involve simple practices, requiring no special skills or training but with proven effectiveness in reducing rates of preventable HAIs [27]. So, it is unreasonable for doctors (or anyone) to claim ignorance or object to being reminded if they fail to observe them even when they perceive no personal risk of infection. There may be valid reasons for disagreement with how policies are deployed, but the patient’s bedside is not the place, and a ward nurse not the person, to resolve them. Will new-found respect for IPC policies and those who seek to implement them persist beyond COVID-19 or will old interprofessional behaviour patterns return?

## Conclusions and a proposal

This study has identified three major perceived interprofessional influences on doctors’ IPC attitudes and behaviours and potential barriers to change:

- preconceived professional stereotypes, including doctors’ aversion to, and nurses’ inflexible application of rules;
- interprofessional differences in beliefs about the importance and effectiveness of IPC; and
- modern versions of the doctor-nurse game in which nurses’ perceived failure to comply with the ‘rules of the game’, when speaking up about IPC, risks a negative response.

Despite different origins, cultures and models of practice, the medical and nursing professions are mutually dependent. Optimal HAI prevention depends on interprofessional collaboration, which is often elusive but critical to patient safety. We argue that it would be enhanced by adherence to basic ethical principles. In the face of ongoing barriers to collaboration, Irvine, et al.proposed a dialogical approach to inter-specialty ethics – an ‘extended collegiality’ [64]. Based on this concept, Clarke et al.proposed a conceptual framework incorporating (i) principles of behaviour; (ii) organisational structures; and (iii) processes to support ethical practices [65]. This framework would provide a suitable ethical basis for interprofessional interactions in the context of hospital IPC:

### Principles

An ethical approach to IPC would include mutual respect and willingness to negotiate interprofessional differences in knowledge and understanding of IPC policies—including which are most important and effective, how to apply them and whether they can be modified without loss of effectiveness. Equitable consultations would be committed to achieving consensus or amicable compromise.

### Structures

Organisational structures to support such an approach would include a coordinated, multidisciplinary system to a) monitor, and feed-back to clinicians, data on the true burden of HAIs; b) prioritise IPC in proportion to the burden; and c) communicate, implement, and evaluate agreed IPC policies. Relevant expertise would be deployed to ensure that policies were: evidence-based (IPC/infectious diseases experts); practicable and context-appropriate (frontline clinicians); and adequately resourced to achieve realistic, enforceable targets (clinical leaders, administrators).

### Processes

The processes required to implement these structures might include: empowerment of staff at all levels to remind others, respectfully, of relevant IPC practices; a mechanism to report unprofessional behaviour, without recrimination, and manage staff, irrespective of status, who consistently fail to comply with agreed policies and/or bully others who attempt to enforce them; multidisciplinary peer-group review of IPC process and outcome measures – e.g. practice audit and HAI surveillance data—and implementation of remedial action if needed.

### Strengths and limitations

A major strength of this study was the seniority and experience of participants and the breadth, variety, and frankness of their views. As a case study, it was appropriately limited to one hospital, but this could be seen to limit its applicability to other settings. However, our experience and the published literature suggest that participants’ perceptions were consistent with those of senior clinicians elsewhere.

## Data Availability

The datasets generated and/or analysed during this study are not publicly available because participants were recruited on the understanding that their comments would remain confidential. However, deidentified extracts of interview transcripts are available from the corresponding author on reasonable request.

## Abbreviations

AMR: Antimicrobial resistance
EID: Emerging infectious disease
EVD: Ebola virus disease
ICU: Intensive care unit
IPC: Infection prevention and control
HAI: Healthcare-associated infection
HCW: Healthcare worker
MERS: Middle Eastern respiratory syndrome
PPE: Personal protective equipment
SARS: Severe acute respiratory syndrome
CNC: Clinical nurse consultant
MDD: Medical divisional director
MUD: Medical unit director
NDD: Nursing divisional director
SMC: Senior medical consultant
VMO: Visiting medical officer
